# Endophilin B2 promotes inner mitochondrial membrane degradation by forming heterodimers with Endophilin B1 during mitophagy

**DOI:** 10.1038/srep25153

**Published:** 2016-04-26

**Authors:** Yi-Han Wang, Jiu-Qiang Wang, Qiaochu Wang, Yun Wang, Caixia Guo, Quan Chen, Tuanyao Chai, Tie-Shan Tang

**Affiliations:** 1 University of Chinese Academy of Sciences, Beijing 100049, China; 2 State Key Laboratory of Membrane Biology, Institute of Zoology, Chinese Academy of Sciences, Beijing 100101, China; 3 Key Laboratory of Genomic and Precision Medicine, China Gastrointestinal Cancer Research Center, Beijing Institute of Genomics, Chinese Academy of Sciences, Beijing 100101, China

## Abstract

Mitochondrial sequestration by autophagosomes is a key step in mitophagy while the mechanisms mediating this process are not fully understood. It has been reported that Endophilin B1 (EB1) promotes mitochondrial sequestration by binding and shaping membrane. However, the role of EB1 homolog Endophilin B2 (EB2) in mitophagy remains unclear. Here we report that EB2 plays an indispensable role in mitochondria sequestration and inner mitochondrial membrane (IMM) protein degradation during mitophagy. Similar to EB1, EB2 aggregates into foci and then translocates to damaged mitochondria. Loss of either EB2 and/or EB1 significantly enervates the foci translocation to fragmented mitochondria and IMM degradation, and the EB1/EB2 heterodimer formed by EB1/EB2 interaction promotes the above process. We noticed that, it is the dimer domain of EB2 but not that of EB1 mediating the heterodimer formation, manifesting the importance of EB2 in mitophagy. Furthermore, we demonstrate that the EB foci formation is closely regulated by the PINK1-Parkin signaling pathway. From these results, we propose that EB1/EB2 heterodimers may serve as linkers between damaged mitochondria and phagophores during mitophagy.

Regulation of mitochondria plays a central role in cellular metabolism. Mitochondrial dysfunction has been linked to multiple human diseases, including heart failure^1^, aging^2^ and neurodegenerative disorders^3^. Under stress, damaged mitochondria are removed by autophagy after activation of PTEN-induced putative kinase 1 (PINK1) and E3 ubiquitin ligase Parkin. After loss of mitochondrial membrane potential, PINK1 is stabilized on mitochondria and recruits Parkin and other effectors^4^. Damaged mitochondria are then sequestered by autophagosomes and finally degraded by lysosomes^5^. The membranes of autophagosomes are derived from various membrane sources, and subsequent processes involving autophagosomes have been shown to be associated with the Beclin-1-PI3KC3 complex^6^. The protein Endophilin B1 (EB1) has been reported to interact with Beclin-1 regulating autophagosome biogenesis^7^ and associate with mitochondrial membrane promoting mitochondrial degradation^8^.

Endophilin B1, also known as Bif-1 (Bax Interacting Factor) and SH3GLB1, is a member of the Endophilin B (EB) family. EB1 was first cloned by using a two-hybrid mating strategy as an interacting partner of human Bax^9^. The interaction between EB1 and Bax undergoes time-dependent changes and promotes conformational changes in Bax leading to oligomerization on the outer mitochondrial membrane (OMM). These changes result in increased OMM permeability during apoptosis^10^. Like other Endophilin family proteins, EB1 contains both Bin/Amphiphysin/Rvs (BAR) and Src-homology 3 (SH3) domains. BAR domains are able to induce and stabilize membrane curvature^11^, while SH3 domains, as protein recognition modules, are able to accommodate a variety of peptide ligands through specific ligand-binding site^12^. Owing to this specialized structure, EB1 is able to promote the formation of a tubular membrane structure *in vitro*^13^. Consistent with these finding, EB1 is required to maintain mitochondrial morphology^14^, suggesting that a part of EB1 located in mitochondria participates in the maintenance of the mitochondrial membrane structure. Moreover, EB1 has been shown to be capable of binding Beclin-1 through UVRAG together with PI3KC3, resulting in the formation of the UVRAG-Beclin-1-PI3KC3 complex and then inducing the generation of Golgi sourced autophagosome during autophagy^7^,^15^. Furthermore, owing to its ability to bind to the membrane, EB1 may promote the sequestration of damaged mitochondria by bridging the gap between autophagosome and damaged mitochondria^8^.

As a new member of the EB family, Endophilin B2 (EB2) is cloned following two-hybrid when EB1 is used as bait. It has been found that EB2 has a specific function in cytoskeletal architecture^16^. Interestingly, EB2 is suspected to be involved in Down syndrome in which EB1 is not involved^17^, suggesting that EB2 may contribute to some disease regulations beyond EB1. Due to high degree of similarity in primary sequence and structure compared with EB1 (ref. 9), EB2 is recognized as the best candidate to functionally compensate for EB1.

As a dimeric molecule, BAR domains of Endophilin facilitate its dimerization controlling membrane curvature. Among Endophilin A family proteins, which have high similarities with EB family proteins, loss of dimerization capacity in Endophilin A1 interrupts its interaction with poly-l-proline and impairs tubular membrane structures formation in normal conditions^18^, suggesting that dimerization may facilitate some dynamic processes such as membrane curvature and effectors binding. Among EB family proteins, BAR domain can also regulate liposome tubulation activity of EB1 by dimerization *in vitro*^13^. Interestingly, the BAR domain in EB1 and EB2 is required for both heterodimer and homodimer formation^9^. Although specific domain mediating dimerization in BAR domain is found, it is unclear how the different dimerizations are regulated respectively. Also, it remains to be determined whether EB2 is required via heterodimerization with EB1 in these described functions of EB1.

In this study, we show that EB2 plays an equal important role in sequestration of the depolarized mitochondria and IMM protein degradation as EB1. We have found that loss of EB2 enervates foci translocation to damaged mitochondria and IMM degradation to the same extent as loss of EB1 does. Heterodimers of EB1 and EB2 aggregate into foci, translocate to mitochondrial fragment, and promote IMM degradation, indicating that EB2 is essential for mitochondria sequestration and mitopahgy. Interestingly, the dimer domain of EB2, but not that of EB1, stabilizes EB1/EB2 heterodimers which are required for efficient mitochondria sequestration and IMM degradation.

## Results

### EB2 translocates to mitochondria during mitophagy

**Figure 1:**
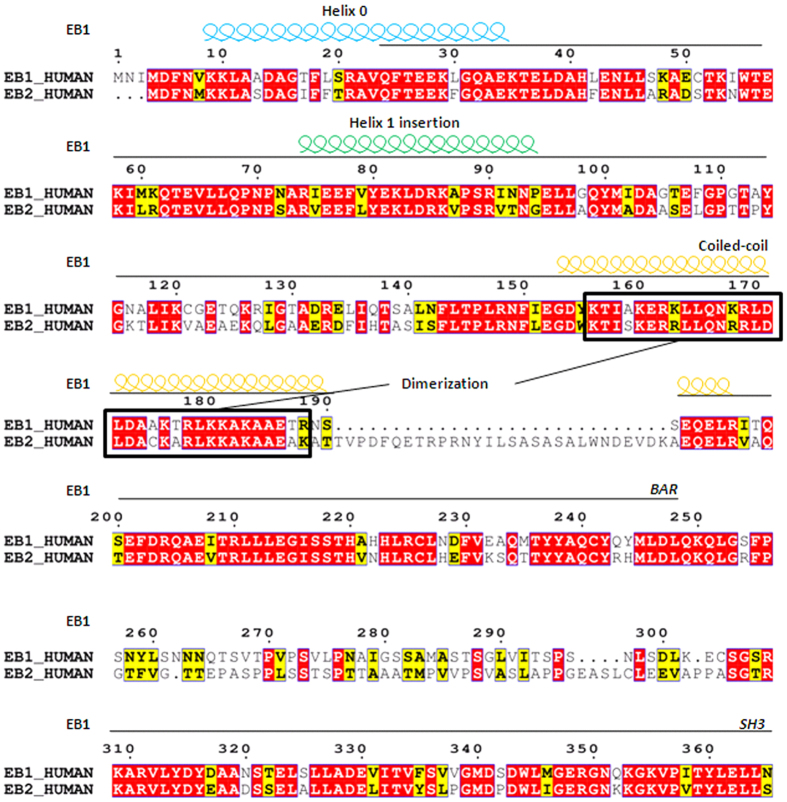
Schematic alignment of the amino acid sequences of human EB1 and EB2. Alignment of protein sequences is exhibited using ESPript 3.0. Conserved residues (red) and similar residues (yellow) are indicated. The amphipathic helix 0 (light blue spirals) that precedes the BAR domain, and the helix 1 insertion (green spirals) were conserved between EB1 and EB2, whereas the coiled-coil domains (yellow spirals) were not conserved. Dimer motifs in the coiled-coil domain are marked with black boxes.

**Figure 2:**
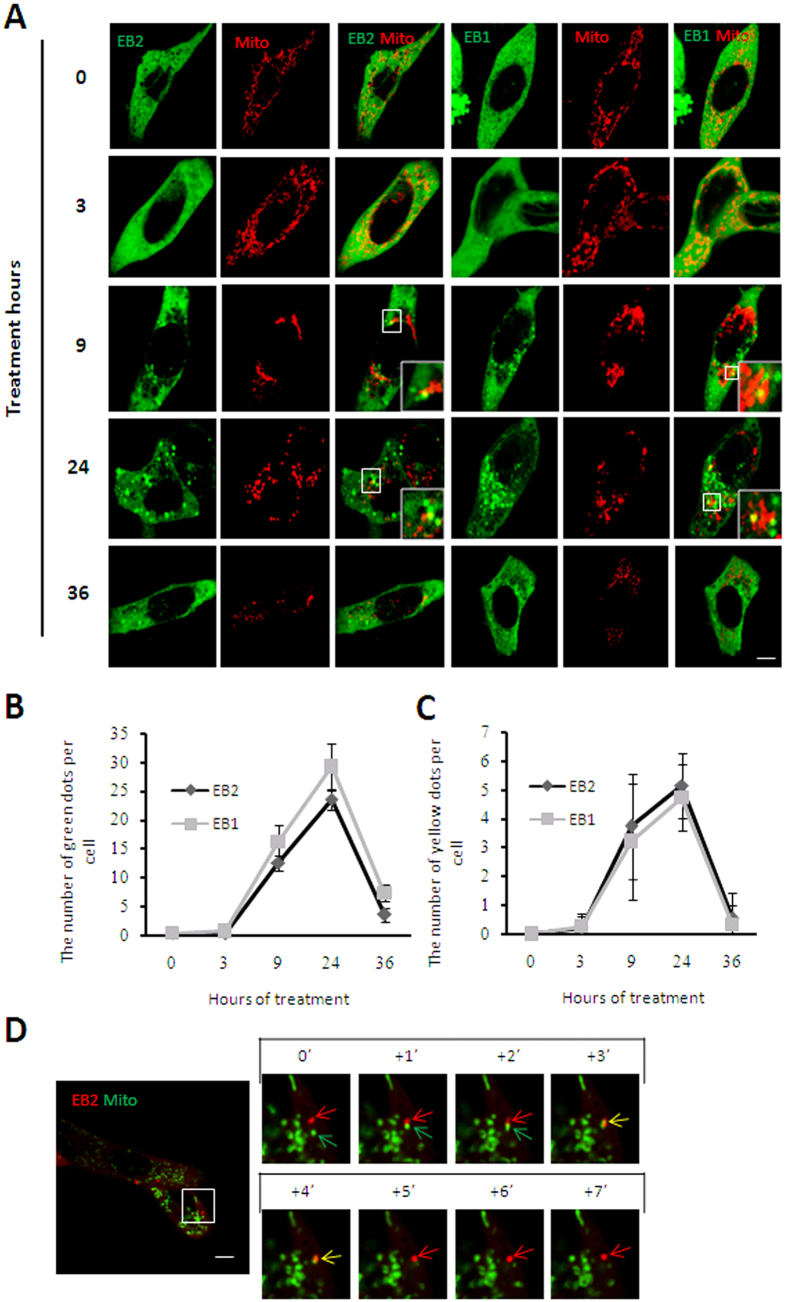
EB2 translocates to mitochondria under mitophagic stress. (**A**–**C**) EB2 aggregated into foci and partially translocated to mitochondria in response to CCCP treatment. HeLa (Parkin-flag expressed) cells expressing mitochondria-DsRed (red) and CFP-EB1 or CFP-EB2 (green) were treated with 20 μM CCCP for the indicated times and analyzed using confocal laser scanning microscope. Magnified images are shown in the insets. Scale bar, 5 μm. The number of GFP positive dots per cell and the number of co-localization with mitochondria (the number of yellow dots) per cell are shown in panels (**B**,**C**), respectively (n = 19, mean ± SD). (**D**) Single foci of EB2 translocated to fragmented mitochondria under CCCP treatment. HeLa (Parkin-flag expressed) cells co-expressing DsRed-EB2 (red) and mitochondria-GFP (green) were treated with 20 μM CCCP for 12 h and then analyzed by time-lapse fluorescent microscopy at 1-s intervals. Red, green and yellow arrows indicate EB2, fragmented mitochondria, and double-positive signals, respectively.

Previous studies revealed that EB1 diffuses in cytoplasm and does not co-localize with mitochondria under basal conditions while aggregates into foci and translocates to mitochondria during mitophagy^8^,^9^. Based on the high homology of EB1 and EB2 (Fig. 1), we speculated that EB2 might also be involved in the process of mitophagy. In response to Carbonylcyanidemchlorophenylhydrazone (CCCP) treatment, EB1 aggregated into foci with the passage of time (Fig. 2A, columns 4^th^–6^th^) in HeLa (Parkin-flag expressed) cells, in which was authenticated (Figure S1). Similar to EB1, EB2 was also distributed evenly in cytoplasm before CCCP treatment (Fig. 2A, row 1^st^, columns 1^st^–3^rd^). During treatment with CCCP, EB2 showed dynamic formation of foci structures and then partially translocated to the mitochondria at almost the same time as EB1 (Fig. 2A, columns 1^st^–3^rd^; Fig. 2D). In the middle stage of mitophagy, mitochondria gathered around the peri-nuclear region, and an increasing number of EB1 and EB2 foci were observed to be co-localized with mitochondria (Fig. 2A). During the late stages of mitophagy, the number of EB1/EB2 foci and the percentages of co-localization with mitochondria decreased (Fig. 2A). To determine the differences between EB1 and EB2 dynamics, we counted the numbers of foci of EB1, EB2 and that co-localizated with mitochondria at indicated time point (Fig. 2B,C). Statistical analysis revealed that CCCP induced similar increases in the numbers of EB1 and EB2 foci and that translocations to fragmented mitochondria in HeLa (Parkin-flag expressed) cells (Fig. 2B,C). Moreover, no more cleavage of Caspase-3 was observed than basal condition after 24 h CCCP treatment in EB2 overexpressing HeLa (Parkin-flag expressed) cells (Figure S2), suggesting that CCCP treatment did not induce apoptosis when EB2 was overexpressed. Thus, EB2 exhibited similar temporal-spatial dynamics as EB1 during mitochondrial stress.

### EB2 promoted IMM protein degradation by cooperating with EB1

**Figure 3:**
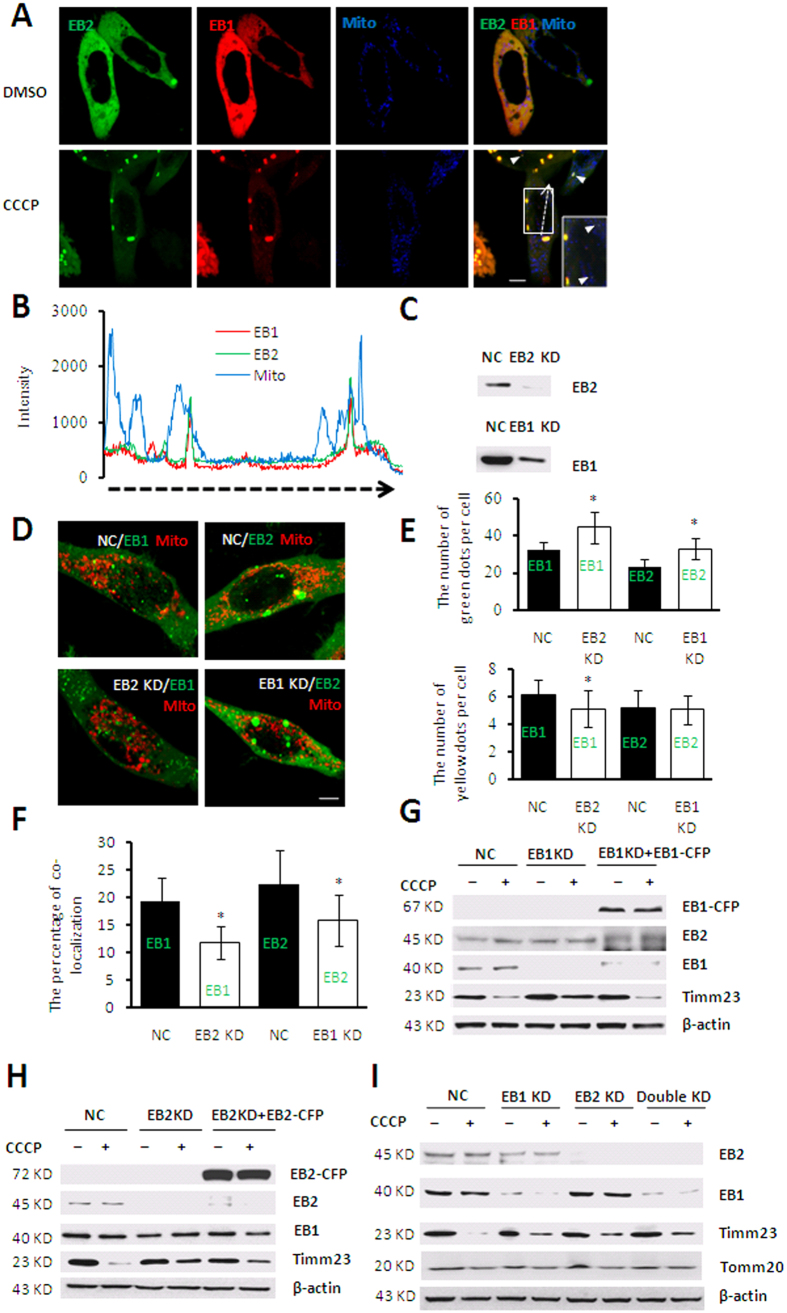
EB2 promotes Timm23 degradation by cooperating with EB1 in response to CCCP treatment. (**A**,**B**) Only EB2-positive EB1 signals were found to co-localize with fragmented mitochondria. HeLa (Parkin-flag expressed) co-expressing YFP-EB2 (green), DsRed-EB1 (red) and mitochondria-CFP (blue) were treated with 20 μM CCCP or DMSO for 24 h. Triple-positive signals are indicated by arrowheads. Magnified images are shown in the insets. Scale bar, 5 μm. The fluorescence intensities along the dotted arrow were quantified using NIS-Elements AR-Analysis software and presented in panel (**B**). The length of horizontal dotted arrow in panel B represents distance of dotted arrow in panel (**A**). (**C**) Low expression of EB1 and EB2 in EB1 and EB2 knockdown cell lines were confirmed by western blotting respectively. (**D**–**F**) Loss of one of EB protein altered the number of foci and percentages of translocation to mitochondria of the other EB proteins in response to CCCP treatment. Cells co-expressing with either CFP-EB1 (green) or CFP-EB2 (green) and mitochondria-DsRed (red) were treated with 20 μM CCCP for 24 h. Scale bar, 5 μm. The number of green and yellow dots per cell and percentages of co-localization with damaged mitochondria (the number of yellow dots/green dots) are shown in panels E and F, respectively. Statistical significance was determined using t tests (n = 19, mean ± SD, *P < 0.05). (**G**–**I**) Knocking down of EB2 or EB1 alleviated Timm23 degradation in response to CCCP treatment. The cells were treated with 20 μM CCCP for 24 h and subjected to immunoblot analysis using the indicated antibodies. NC: negative control, EB1 KD: EB1 knockdown cells, EB2 KD: EB2 knockdown cells, Double KD: EB1- and EB2-knockdown cells, EB1 KD + EB1: CFP-EB1-expressing cells with knockdown of endogenous EB1, EB2 KD + EB2: CFP-EB2-expressing cells with knockdown of endogenous EB2.

To further determine whether EB1 and EB2 function through the same mechanism during mitophagy, we co-expressed EB1 and EB2 in HeLa (parkin-flag expressed) cells. Fluorescent image analysis showed that EB2 synchronized with EB1 in the process of foci formation and co-localized with EB1 during mitophagy (Fig. 3A). Moreover, Some EB1/EB2 double-positive signals were associated with fragmented mitochondria, whereas EB1 or EB2 single-positive signals were not (Fig. 3B). In order to further determine the relationship between EB1 and EB2 in mitophagy, we established EB1 and EB2 knockdown (KD) cells using a short hairpin RNA (shRNA)-activated gene-silencing system. HeLa (Parkin-flag expressed) cells were transfected with shRNAs targeting either an untranslated region (UTR) of the human *EB2* gene (EB2 KD cells) or a coding DNA sequence (CDS) of the human *EB1* gene (EB1 KD cells), and dramatic reductions of EB1 and EB2 protein expression level were achieved (Fig. 3C). To examine the relative role of EB1 and EB2 during mitophagy, the number of foci and that translocated to the mitochondria were determined in EB1 and EB2 KD cells. The results showed that the number of EB1 foci increased significantly after loss of EB2, whereas the percentage of EB1 foci translocated to damaged mitochondria decreased significantly (Fig. 3D,F). Similarly, results for EB2 were also obtained in CCCP-treated EB1 KD cells, though the co-localized foci of EB2 and mitochondria accumulated similar number in negative control (NC) and EB1 KD cells (Fig. 3D,F). These results indicated that EB2 and EB1 compensated for the loss of the other protein during the formation of foci, which is consistent with a previous report^19^. Intriguingly, our results further demonstrated that both EB1 and EB2 are indispensible for EB foci mitochondrial translocation.

To further analyze the effects of co-localization of EB2 and EB1 on mitochondria during mitophagy, we knocked down EB1 and EB2 separately or together. As previously reported, loss of EB1 abrogated the degradation of IMM protein COX IV and mitochondria matrix protein Hsp60 while it had little effect on degradation of outer mitochondria membrane (OMM) protein Tomm20 during mitophagy^20^. In agreement with these results, Timm23 reduction was alleviated by loss of EB1 in response to CCCP treatment, but not Tomm20 (Fig. 3G). Similar results were also found in EB2 KD cells (Fig. 3H). Moreover, our results showed that loss of either one or both had an equal extent of alleviation on Timm23 degradation during mitophagy (Fig. 3I), demonstrating that EB2 act same important role as EB1 for Timm23 degradation rather than simply compensate it. In addition, the degradation of Timm23 was not abrogated completely, suggesting that other signal pathways may mediate mitophagy independent of EB1 and EB2. The degradation of Tomm20 is slower than that of Timm23, implying that the degradation of Tomm20 and Timm23 may involve different pathways. These results indicated that cooperation of EB1 and EB2, rather than EB1 or EB2 alone, promoted IMM degradation.

### EB2 mediated IMM protein degradation by promoting heterodimer translocation

**Figure 4:**
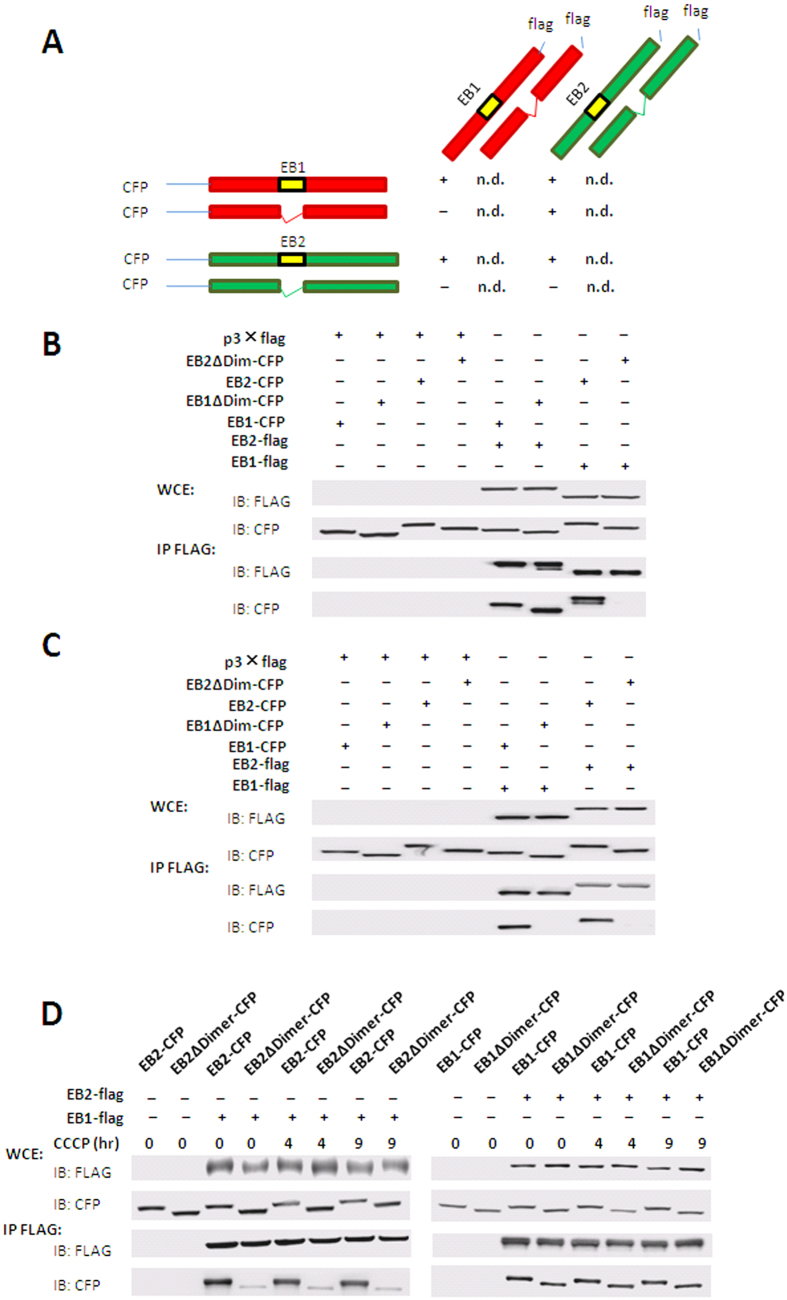
The dimer domain of EB2 mediates the heterodimerization of EB1 and EB2. (**A**) Schematic diagram summarizing interactions between EB1 and EB2. 293T cells were co-transfected with full-length and truncated EB1 or EB2 tagged with flag or CFP. Cell lysates were then subjected to immunoprecipitation with anti-flag monoclonal antibodies and then analyzed by immunoblotting. Immunoprecipitation results were marked by “+”, “−” and “n.d.”, which means “interaction”, “no interaction”, and “not determined”. (**B**) The roles of dimer domains of EB1 and EB2 in hetero-interaction between EB2 and EB1. (**C**) The roles of dimer domains of EB1 and EB2 in self-interaction of EB1 and EB2. (**D**) Effects of CCCP treatment on the hetero-interactions between EB1 and EB2.

It has been known that EB1 induces autophagy via dimerization but whether such dimerization affects mitophagy remains unclear. Based on our result that either single type of EB protein alone could not mediate IMM protein degradation, we suspected that this process might be mediated by the EB1/EB2 heterodimers. To test this hypothesis, we constructed vectors encoding truncated EB1 and EB2 which could not mediate their dimerization. Amino acid residues 153–185 in EB2 (dimer domain of EB2) and 156–188 in EB1 (dimer domain of EB1) in their respective BAR domains have been reported to mediate both heterodimer and homodimer formation in yeast. To investigate which truncation might impair the interaction of EB1 and EB2, we deleted the dimer domains of EB1 and EB2, and then analyzed their interaction by co-immunoprecipitation. As our data shown, deletion of the dimer domain of EB2 abolished its interaction with both EB1 (Fig. 4B) and EB2 (Fig. 4C); whereas deletion of the dimer domain of EB1 did not affect its interaction with EB2 (Fig. 4B), but ravaged its interaction with EB1 (Fig. 4C). Therefore, the dimer domain of EB2 was essential for its heterodimerization and homodimerization while the dimer domain of EB1 was only essential for its homodimerization (Fig. 4A), thus allowing us to identify both heterodimers and monomers. Moreover, in agreement with previous data in yeast^9^, the EB1-EB2 interaction detected in HEK 293T cells was not altered in response to CCCP treatment (Fig. 4D), suggesting that their foci formation was not relied on their heterodimerization.

**Figure 5:**
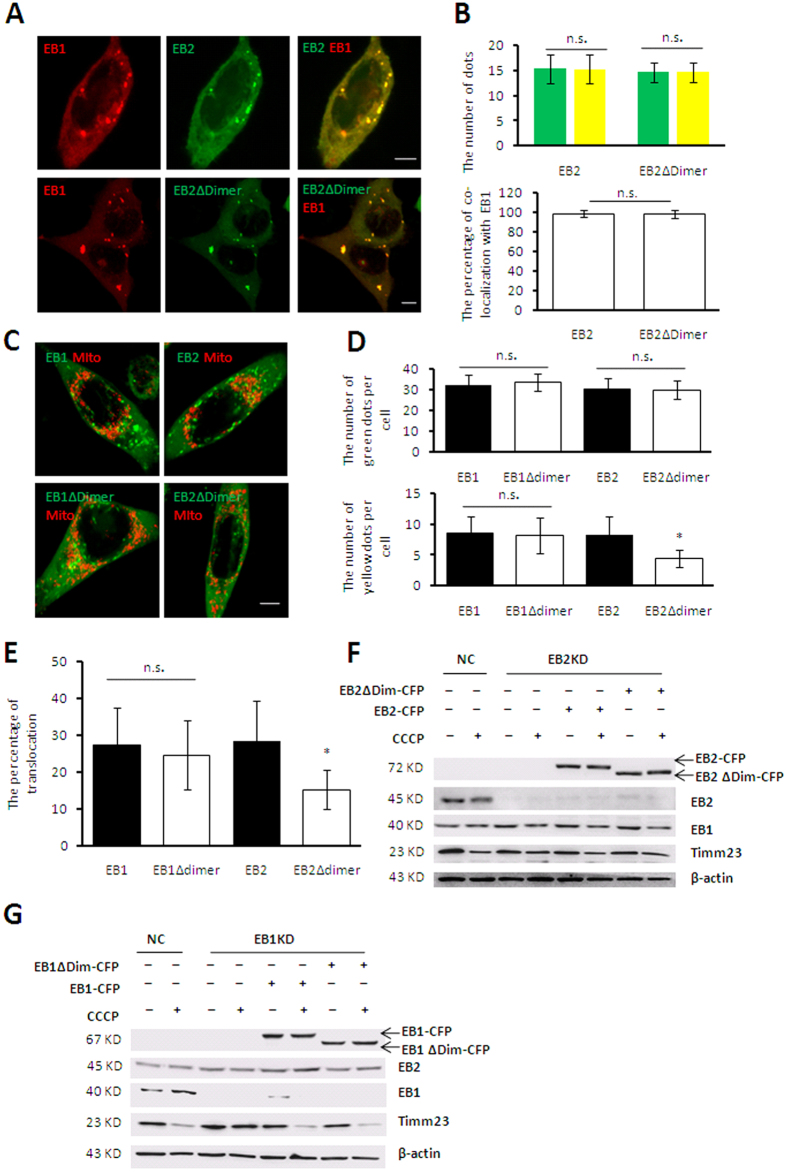
The heterodimer of EB1 and EB2 facilitates IMM protein degradation by promoting foci translocation to mitochondria. (**A**,**B**) Co-localization of EB1 and EB2 foci was not mediated by the dimer domain of EB2. HeLa (Parkin-flag expressed) cells co-expressing with RFP-EB1 (red) and CFP-EB2 or CFP-EB2Δdimer (green) were treated with 20 μM CCCP for 20 h. Scale bar, 5 μm. Co-localization of RFP-EB1 with EB2 or EB2Δdimer is shown in the panel (**A**) and the number of dots (green column represent green dots and yellow column represent yellow dots) and the percentage of co-localization is shown in panel (**B**). Statistical significance was determined using t tests (n = 19, mean ± SD). n.s. represents for no significant difference. (**C–E**) Loss of the EB2 dimer domain reduced the translocation of EB2 foci. HeLa (Parkin-flag expressed) cells expressing the full-length or truncated EB1 or EB2 were treated with 20 μM CCCP for 20 h. Images from microscopic analysis are shown in panel (**A**). Scale bar, 5 μm. The number of green dots and yellow dots and the percentages of foci translocation to mitochondria are shown in panels (**D,E**), respectively. Statistical significance was determined using t test (n = 19, mean ± SD, *P < 0.05). (**F–G**) Disruption of heterodimerization alleviated Timm23 degradation in response to CCCP treatment. Negative control (NC), EB1-knockdown (EB1 KD) and EB2-knockdown (EB2 KD) cells expressing the indicated proteins were treated with or without 20 μM CCCP for 20 h as shown. Cells were then subjected to immunoblot analysis using the indicated antibodies.

To further investigate whether mitochondrial sequestration depend on the heterodimers of EB1 and EB2 or separated EB1 and EB2 in mitophagy after both EB1 and EB2 was demonstrated indispensible, the proteins with deletion of the dimer domains (EB1Δdimer and EB2Δdimer) were used for indicating heterodimers and EB2 monomers respectively in EB1 KD and EB2 KD cells. Our results showed that the EB2 monomer was able to co-localize with EB1 (Fig. 5A,B), suggesting that the interaction between EB1 and EB2 did not affect their co-localization. Moreover, the EB2 monomer formed a similar number of foci as the heterodimer (Fig. 5C,D), indicating that the formation of EB2 foci was not mediated by its interaction with EB1. However, EB1 and EB2 double positive foci accumulated into lower number and larger dots than single positive foci of EB1 or EB2 in Fig. 1A (Fig. 5A, upper panel), probably due to high interaction between EB1 and EB2. Furthermore, the EB2 monomer exhibited a significantly lower rate of translocation to fragmented mitochondria than the heterodimer (Fig. 5C,E), demonstrating a stimulative effect of heterodimer on foci translocation. Moreover, EB2 overexpression could rescue the alleviation of Timm23 degradation, whereas the EB2 monomer could not in CCCP-treated EB2 KD cells. In contrast, the degradation of Timm23 was restored when overexpressing EB1 or heterodimer in EB1 KD cells (Fig. 5F,G) during mitophagy. Taken these results together, we concluded that EB2 promoted the translocation of foci to fragmented mitochondria and IMM degradation via forming heterodimer with EB1.

### The formation of foci structures was regulated by the PINK1-Parkin signaling pathway

**Figure 6:**
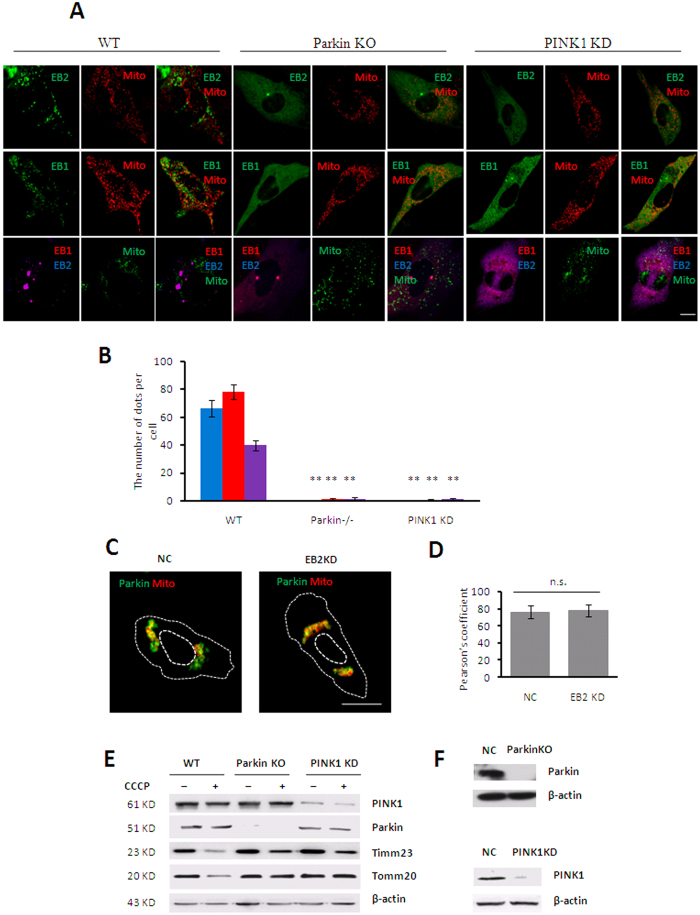
Foci formations of EB1 and EB2 were regulated by PINK1-Parkin. (**A,B**) The formation of EB family protein foci was abolished in Pink1- and Parkin-deficient cells. The indicated proteins were co-expressed in Parkin^+/+^ (wild type [WT]), Parkin^−/−^ (knockout [KO]), and PINK1-knockdown MEFs, and cells were treated with 30 μM CCCP for 12 h. Microscopic images are shown in panel (**A**), red, blue and purple column represent EB1, EB2 and EB1EB2 respectively. Scale bar, 10 μm. The number of foci is shown in panel (**B**). Statistical significance was determined using t test (n = 19, mean ± SD, **P < 0.01). (C–D) Parkin translocation to fragmented mitochondria in EB2 KD cells in response to CCCP treatment. Parkin-GFP and mitochondria-DsRed were co-expressed in NC and EB2 KD cells, and cells were treated with CCCP for 9 h. Microscopic images are shown in panel (**C**). Scale bar, 10 μm. Histogram shows Pearson’s coefficient between Parkin and damaged mitochondria in WT or EB2 KD cells in panel (**D**). Statistical significance was determined by using t test. n.s. represents for no significant difference. (**E**) Timm23 degradation was significantly limited in Parkin^−/−^ MEFs and PINK1 KD MEFs in response to CCCP treatment. Indicated cells were treated with or without CCCP as shown. Cells were then subjected to immunoblot analysis using the indicated antibodies. (**F**) Parkin and PINK1 expression levels in Parkin^−/−^ and PINK1 KD cells were examined by immunoblotting.

PINK1 and Parkin mediated mitophagy signal pathway has been recognized as a major process associated with mitochondrial clearance^4^,^20^. Based on our result that the formation of EB1/EB2 foci was the first stage of EB1-induced mitophagy, we are wondering whether the foci formation was regulated by the PINK1-Parkin signaling pathway. PINK1- and Parkin-deficient mouse embryonic fibroblasts (MEFs) were utilized to examine the EB1/EB2 foci formation. Our results showed that EB1 and EB2 aggregated into foci and partially translocated to fragmented mitochondria in wild-type MEFs after CCCP treatment, but fewer number of EB1 and EB2 double positive foci were observed, which consistent with results in HeLa (Parkin-flag expressed) cells. In contrast, few EB1 and EB2 proteins aggregated into foci in both Parkin- and PINK1-deficient MEFs (Fig. 6A,B) in mitophagy. Moreover, Parkin translocated to fragmented mitochondria regardless of EB2 expression in response to CCCP treatment (Fig. 6C,D), thus suggesting that EB2 acts downstream of the Parkin signaling pathway. Meanwhile, limited Timm23 degradation was also found in both PINK1- and Parkin-deficient MEFs during mitopahgy (Fig. 6E), whereas degradation of OMM protein Tomm20 was completely abolished in these two MEF lines. Taken together, we concluded that the formation of EB1 and EB2 foci and the subsequent IMM degradation involved PINK1-Parkin signaling pathway.

## Disscusion

**Figure 7:**
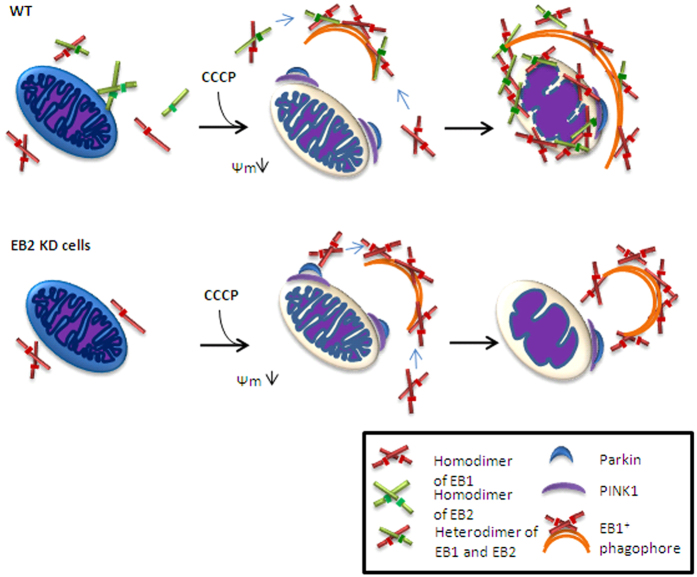
Hypothetical model of EB2-mediated mitochondrial sequestration. Mitochondria and different forms of EB1 and EB2 diffused in cytoplasm are shown. In response to mitochondrial damage, Parkin was recruited by PINK1 on OMM. Subsequently, heterodimers in the cytoplasm aggregated into foci and fused with the EB1 positive phagophore to induce mitochondria sequestration. Without heterodimerization, homodimers aggregated into more foci to compensate for loss of heterodimers, but mitochondria sequestration and IMM degradation were significantly limited.

Mitophagy plays a key role in the clearance of damaged mitochondria. However, the molecular mechanisms of mitochondrial sequestration by autophagosome after ubiquitination of OMM proteins are not fully understood. In this study, we found, for the first time, that EB2 was involved in mitophagy through formation of heterodimers with EB1. Moreover, the EB1/EB2 heterodimers, but not homodimers, promoted IMM degradation via their foci translocation to damaged mitochondria (Fig. 7). Interestingly, dimer domain of EB2 but not that of EB1, is important for heterodimerization. All these data demonstrate the indispensible role of EB2 in mitochondria sequestration and IMM degradation in mitophagy.

In previous studies, mitochondria have been shown to be sequestered by autophagosomes in mitophagy. One study^21^ showed that Parkin was recruited to damaged mitochondria by PINK1 and then ubiquitinated VDAC1 to recruit p62 which mediated mitochondrial sequestration by interacting with LC3. Another study^22^ showed that PINK1 recruited NDP52 and optineurin to damaged mitochondria and then recruited the autophagy factor ULK1 to mediate autophagosome formation. However, after damaged mitochondria are recognized, related proteins are needed to bind to and shape the membrane^8^. EB1 has been reported to be associated with Ptdlns3P, directly inducing membrane tubulation^13^. In addition, EB1 is also considered as an effector in autophagosome formation^7^. Thus, EB1 may act as a bridge between damaged mitochondria and autophagosomes^8^. Consistently, we discovered that EB1 aggregated into foci that partially translocated to fragmented mitochondria, promoting degradation of the IMM protein Timm23. Moreover, these foci are known to co-localize with autophagosomes^20^. Thus, these results provide insights into the role of EB1 in damaged mitochondria and autophgosomes. Notably, Parkin translocation is observed, regardless of the formation of EB1 foci or the translocation of EB1 foci in response to CCCP treatment^20^. Our data revealed that the formation of such foci was abolished in the absence of Parkin or PINK1 (Fig. 6), indicating that Parkin translocation initiated the regulatory functions of EB1 following specific events.

EB2 is a homolog of EB1 and shares a similar structure with EB1. However, its function in mitophagy remains unclear. Here, we reported that EB2 played an important role in mitophagy. During mitophagy, EB2 aggregated into foci, colocalizing with EB1 and these EB1/EB2 foci partially translocated to damaged mitochondria in a time-dependent manner. According to previous results, EB1 foci colocalize with mATG9 during starvation and mATG9-containing vesicles are recognized as a source of membranes of autophagosome^23^,^24^. Thus, it is possible that EB2 foci located in cytoplasm are involved in the expansion of autophagosomes. Our data showed that loss of EB2 reduced IMM degradation in HeLa (Parkin-flag expressed) cells, regardless of EB1 expression or not. Therefore, unlike previous studies which suggested that EB2 functions to compensate EB1 in mitophagy, our data support that EB2 is an indispensable protein in mitophagy. Notably, the dimer domain of EB2 is important for the heterodimerization between EB1 and EB2, and this may be due to the extra sequence in EB2 (i.e., the coiled-coil domain, as shown in Fig. 1). As previously reported^25^, PINK1 is stabilized on the OMM and then recruits other effectors to mediate mitophagy. Among these different effectors, Parkin has been shown to have an important role through interactions with different signal proteins^26^. In our study, the formation of EB2 foci was shown to be regulated by Parkin and PINK1 and loss of PINK1 and Parkin blocked IMM degradation. Thus, these data suggested that OMM proteins were ubiquitinated by Parkin, triggering the formation of EB2 foci which initiated downstream mitochondrial sequestration.

The importance of heterodimerization during mitochondria sequestration and IMM degradation has also been demonstrated in our study. EB1 and EB2 were shown to form homodimers and heterodimers under normal conditions, and both proteins could aggregate into foci during mitophagy. Although we could not quantitative analysis constituents in foci, heterodimer was shown to be a key part. Without heterodimerization, the translocation of foci to damaged mitochondria and IMM degradation were significantly reduced in response to CCCP treatment. Therefore, we speculated that EB1 homodimer might be involved in autophgosomes expansion instead of mitochondrial sequestration, whereas EB heterodimer is responsible for mitochondrial sequestration. However, it is unclear at this point that whether heterodimers participate in the tubulation of the membrane and expansion of autophgosomes.

In conclusion, our results showed that EB2 played an important role in mitophagy. EB2 promoted the translocation of foci by forming heterodimers with EB1 and subsequently facilitated IMM degradation. In addition, the formation of EB1 and EB2 foci initiated the mitochondrial sequestration. Finally, our study also showed that PINK1 and Parkin played an important role in the formation of EB foci and damaged mitochondria sequestration during mitophagy.

## Materials and Methods

### Plasmids

The cDNA encoding for mouse *EB1* and human *EB2* were cloned into the pECFP-C1, pDsred-C1, pEYFP-C1 and p3 × flag-myc-cmv-26 vectors. EB1 (Δ156–188) and EB2 (Δ153–185) were cloned into the pECFP-C1 and pDsred-C1vectors, respectively. The cDNA encoding for human cytochrome c oxidase subunit VIII (1–87) was cloned into pECFP-N1. Mitochondria-GFP-N1 and mitochondria-RFP-N1 were constructed as previously described^27^.

### Antibodies and regents

The following primary antibodies were used in this study: anti-GFP rabbit polyclonal antibodies (1:5000; Abmart), anti-flag mouse monoclonal antibody (1:5000; Beyotime), anti-Tomm20 rabbit polyclonal antibodies (1:2000; Proteintech, 11802-1-AP) were employed to detect mouse Tomm20, purified mouse anti-Timm23 antibodies (1:1000; BD Biosciences, 611223) were used to detect human and mouse Timm23, anti-β-actin mouse monoclonal antibodies (1:100000; Abmart, 224175) were used to detect human and mouse β-actin and anti-Bif-1 mouse monoclonal antibodies (1:1000; Imgenex, IMG-265A) were used to detect both human and mouse EB1 and human EB2. The following Secondary antibodies were used in this study: Horseradish peroxidase (HRP)-conjugated Affinipure goat anti-rabbit (1:10000; Jackson) and HRP-conjugated Affinipure goat anti-mouse (1:10000; Jackson). CCCP and Puromycin were purchased from Sigma.

### Cell culture, establishment of varied stable cell lines

HeLa, HEK 293T cell lines and MEFs were grown in Dulbecco’s modified Eagle’s medium (DMEM; Gibco) supplemented with 10% fetal calf serum, 2 mM l-glutamine, 21 mM HEPES, 44 mM NaHCO_3_, at 37 °C in 5% CO_2_.

Authentication testing of HEK 293T and HeLa cell lines have been performed by Shanghai Biowing Applied Biotechnology Co.,Ltd via STR profiling. STR profiles match the standards recommended for HEK 293T and HeLa cell lines authentication^28^.

For the establishment of HeLa (Parkin-flag expressed) stable cell lines, HEK 293T cells were transfected with lentiviral packaging plasmids and pWPXLd-Parkin-flag-Neo. Supernatants were collected after 48 h and filtered to infect HeLa cells. After trypsinization and appropriate dilutions, cells were grown for 2 weeks in culture medium supplemented with 50 μg/mL G418, and single colonies were picked. The efficiencies of expression were determined by western blotting (Figure S1).

For the establishment of EB1 and EB2 KD stable cell lines, HEK 293T cells were transfected with lentiviral packaging plasmids and related shRNA vectors (GV112, Genechem). Supernatants were collected after 48 h and filtered to infect HeLa (Parkin-flag expressed) cells. After trypsinization and appropriate dilutions, cells were grown for 2 weeks in culture medium supplemented with 2 μg/mL puromysin, and single colonies were picked. The efficiencies of EB1 and EB2 KD were determined by western blotting (Fig. 3C).

The shRNA target sequences were as follows:

EB2 5′-GCCCGTCATTCAGCTATTAAACTC-3′ and

EB1 5′-TTCAACAAGTGGCCTAGTAAT-3′

For the establishment of the PINK1 KD MEFs, HEK 293T cells were transfected with lentiviral packing plasmids and the related shRNA vector (LV2, GenePharm). Supernatant was collected after 48 hrs and filtered to infect MEF cells. The stable KD MEFs were established as above. The efficiency of PINK1 KD was determined by western blotting.

The shRNA target sequence: 5′-CCTGGCTGACTATCCTGATAT-3′

Parkin−/− mouse were obtained from Jackson Lab (B6.129S4-Park2tm1Shn/J). MEFs were isolated from day 13.5 embryos as described^29^,^30^. The efficiency of Parkin knocking out was demonstrated by western blotting.

### Ethics statement

Our works involving mouse embryo collection and MEF cell culture were in full compliance with the Regulations for the Care and Use of Laboratory Animals by the Ministry of Science and Technology of China, and with the Institute of Zoology’s Guidelines for the Care and Use of Laboratory Animals. The experimental protocol was approved by the Animal Care and Use Committee at the Institute of Zoology, Chinese Academy of Sciences.

### Plasmid and shRNA transfection

For transient expression, cells grown at 70% confluence were transfected with polyetherimide (Polysciences) and plasmid DNA or shRNA mixture according to the manufacturer’s instructions. Cells were used between 20-and 24 h post-transfection.

### Immunoprecipitation and immunoblottings

For co-immunoprecipitation^29^, transfected HeLa cells (85–90% confluence) in 10 cm-dishes were used. Cells were washed twice with ice-cold phosphate-buffered saline (PBS) and lysed in 1 mL of NETN buffer (100 mM NaCl, 20 mM Tris-Cl [pH 8.0], 0.5 mM EDTA, 0.5% [v/v] Nonidet P-40 [NP-40]) complemented with proteinase inhibitor (Amresco). After 20 min, lysate were centrifuged for 20 min at 13000× g to collect supernatants. Then, 70 μL of each lysate was used for determination of the signals in the input. Twenty-five microliters of M2 beads (Sigma) were washed twice with NETN buffer and then incubated with supernatants. Beads were washed four times with 500 μL of high-salt NETN buffer (300 mM NaCl, 20 mM Tris-Cl [pH 8.0], 0.5 mM EDTA, 0.5% [v/v] NP-40) and washed once with 500 μL of NETN buffer. Beads were then resuspended in 30 μL SDS loading buffer followed by SDS- PAGE and immunoblotting. All steps were performed at 4 °C.

### Living cell imaging

Cells were cultured on the coverglass bottom 35 mm dish, and imaged by using confocal laser scanning microscope (NiKon N-SIM). Images stacks comprised several focal planes for different cells (5 focal planes for HeLa and 1 focal plane for MEFs). For better exhibition of figures, stacks were finally processed for obtaining complete z-projections.

### Statistical analysis

For quantitative analyses of dots (Image J), values were obtained from cells in an experiment and expressed as the mean ± SD. Statistical analyses were exhibited by using student t test. P-values less than 0.05 was considered significant, *P < 0.05 and **P < 0.01 versus the corresponding controls were indicated.

Alignment is performed using ESPript 3.0 as described previously^31^, which is freely available online at http://espript.ibcp.fr/ESPript/ESPript/index.php.

The fluorescence intensities were quantified (Fig. 3B) and Pearson’s coefficient was documented (Fig. 6D) using NIS-Elements AR-Analysis (NiKon N-SIM).

## Additional Information

**How to cite this article**: Wang, Y.-H. *et al*. Endophilin B2 promotes inner mitochondrial membrane degradation by forming heterodimers with Endophilin B1 during mitophagy. *Sci. Rep*. **6**, 25153; doi: 10.1038/srep25153 (2016).

## Supplementary Material

Supplementary Information
