# Supplementary material for: Endophilin B2 promotes inner mitochondrial membrane degradation by forming heterodimers with Endophilin B1 during mitophagy
Source: Sci Rep. 2016 Apr 26;6:25153. doi: 10.1038/srep25153 (PMC4845016; doi:10.1038/srep25153)
Supplement: Supplementary Information [file srep25153-s1.pdf]

Supplemental information

Endophilin B2 promotes inner mitochondrial membrane degradation by forming heterodimers with Endophilin B1 during mitophagy

Yi-Han Wang[1,2,4], Jiu-Qiang Wang[2,4], Qiaochu Wang[2,4], Yun Wang[2], Caixia Guo[3], Quan Chen[2], Tuanyao Chai*[1], Tie-Shan Tang*[2]

[1]University of Chinese Academy of Sciences, Beijing 100049, China
[2]State Key Laboratory of Membrane Biology, Institute of Zoology, Chinese Academy of Sciences, Beijing 100101, China
[3]Key Laboratory of Genomic and Precision Medicine, China Gastrointestinal Cancer Research Center, Beijing Institute of Genomics, Chinese Academy of Sciences, Beijing 100101, China
[4]Equal contribution.
*Correspondence: tangtsh@ioz.ac.cn; tychai@ucas.ac.cn

Figure S1

A

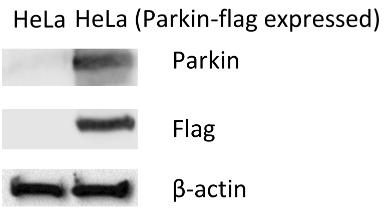

B

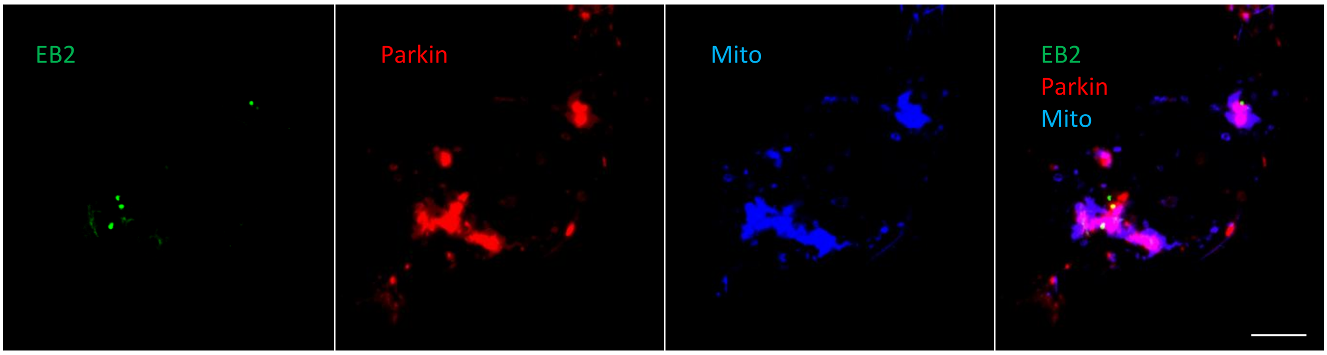

Parkin expression and translocation to damaged mitochondria during CCCP treatment were confirmed in HeLa (Parkin-flag) cells.

(A) Parkin expression in HeLa (Parkin-flag) was performed by western blot using indicated antibodies.

(B) HeLa (Parkin-flag) cell expressing EB2-YFP (green) and Mito-BFP (blue) was treated with CCCP (20 μM) for 9 hours and then subjected to immunostain with flag antibodies (red). Scale bar, 5 μm.

Figure S2

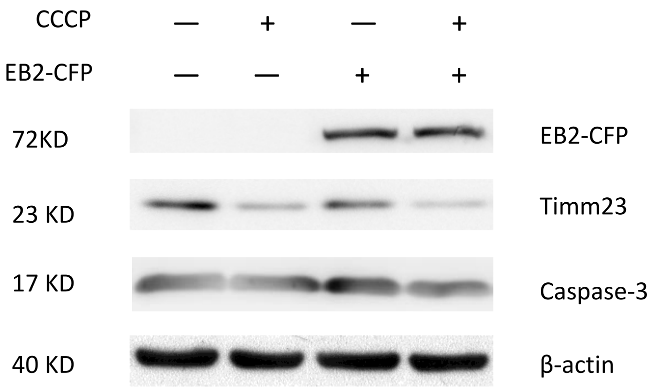

CCCP treatment and EB2 overexpression did not induce caspase-3 cleavage. HeLa (Parkin-flag expressed) expressing EB2-CFP or not were treated with CCCP for 24 h or not as indicated and then subjected to immunoblot analysis by using indicated antibodies.

Supplemental Methods

Immunoblot analyses

Cell lysates were prepared in SDS loading buffer. The following primary antibodies were used: purified mouse anti-Timm23 antibodies (1:1000; BD Biosciences, 611223) , anti-caspase-3, p17 specific rabbit polyclonal antibodies (1:1000; proteintech, 25546-1-AP), anti-Parkin mouse monoclonal antibodies (1:1000; EMD Millipore, 05-882), anti-GFP rabbit polyclonal antibodies (1:5000; Abmart), anti-β-actin mouse monoclonal antibodies (1:100000; Abmart, 224175). The following secondary antibodies were used: Horseradish peroxidase (HRP)-conjugated Affinipure goat anti-rabbit (1:10000; Jackson) and HRP-conjugated Affinipure goat anti-mouse (1:10000; Jackson).

Immunofluorescence microscopy

HeLa (Parkin-flag expressed) cells expressing EB2-YFP and mito-BFP were treated with CCCP for 9h and then fixed in 4% PBS-paraformaldehyde, permeabilized with 0.25% triton x 100 for 10 min, incubated with anti-flag mouse monoclonal antibody (1:5000; Beyotime) against Parkin-flag followed by secondary antibodies conjugated with Alexa 546. Images were obtained from confocal microscope of NiKon N-SIM.
